# Three Cases of a Specific Learning Disorder Diagnosed at the Onset of Type 2 Diabetes

**DOI:** 10.7759/cureus.94757

**Published:** 2025-10-16

**Authors:** Yuriko Watanabe, Hiromune Narusawa, Fumikazu Sano, Hideaki Yagasaki, Yoshimi Kaga

**Affiliations:** 1 Department of Pediatrics, Faculty of Medicine, University of Yamanashi, Yamanashi, JPN

**Keywords:** attention-deficit/hyperactivity disorder (adhd), coordination disorders, dysgraphia, executive dysfunction, neurodevelopmental disorders, obesity, specific learning disorders, type 2 diabetes

## Abstract

In children with type 2 diabetes, obesity is a particularly significant risk factor. Additionally, obesity has been reported to be particularly prevalent in children with neurodevelopmental disorders, attributed to various potential causes. We present three pediatric cases diagnosed with type 2 diabetes, all of whom also had dysgraphia, a specific learning disability, and attention-deficit/hyperactivity disorder (ADHD) with predominantly inattentive symptoms. The cases involved three male children aged 11-14 years. All had a tendency toward obesity since early childhood, had poor weight control, and were later diagnosed with type 2 diabetes. All of them exhibited learning difficulties, and psychological testing revealed coexisting dysgraphia and ADHD. Additionally, Case 1 had autism spectrum disorder, and Cases 2 and 3 had developmental coordination disorder (DCD). Further testing revealed executive function disorders in all cases.

Associations between neurodevelopmental disorders and obesity, and between ADHD and executive dysfunction, have been previously reported. In all cases, it is plausible that obesity progressed and led to the development of type 2 diabetes due to the characteristics of the neurodevelopmental disorders and the associated executive dysfunction. Additionally, the presence of inattentive-type ADHD, impaired visual cognition in Case 1, and DCD in Cases 2 and 3 may have contributed to the development of dysgraphia. While some reports suggest a genetic association between dyslexia and type 2 diabetes, the factors contributing to dysgraphia are diverse, and the genetic link remains unclear. These cases suggest that childhood-onset type 2 diabetes may be associated with neurodevelopmental disorders and executive function deficits. Therefore, clinicians and caregivers should be mindful of the coexistence of neurodevelopmental disorders so that these children receive the appropriate diagnosis and support.

## Introduction

Type 2 diabetes in children is caused by pancreatic beta-cell dysfunction combined with insulin resistance due to obesity, particularly the accumulation of visceral fat, which leads to insufficient insulin action [1]. A family history of diabetes, low or high birthweight, increased growth, and sex hormone secretion during puberty are also listed as risk factors. However, since obesity is present in 80% of children with type 2 diabetes, it is an important risk factor for this condition [1,2].

Types of neurodevelopmental disorders include autism spectrum disorder (ASD), attention-deficit/hyperactivity disorder (ADHD), and specific learning disorders (SLD), which may co-occur. Children with neurodevelopmental disorders have a higher incidence of obesity. For example, one study reported that the risk of obesity was 41.4% higher in individuals with ASD [3]. Another study found that the odds of obesity were approximately 40% higher in children with ADHD than in typically developing children [4]. The causes include abnormal eating behaviors, limited physical activity, genetic predisposition, medication effects, and coexisting sleep disorders [3]. However, the underlying pathophysiology and mechanisms remain unclear. Additionally, types of SLD include dyslexia, dysgraphia, and dyscalculia. Generally, SLD typically manifests as learning difficulties after elementary school enrollment, and most often receives a diagnosis by age 10 at the latest. We report three cases diagnosed with SLD following a type 2 diabetes diagnosis. We identified underlying ADHD with inattentive predominance and/or executive dysfunction in these patients.

## Case presentation

Case 1

An 11-year-old male was diagnosed with ASD at the age of three years owing to a delay in language development. Weight gain was noted during a school health checkup and upon examination at our clinic. His fasting blood glucose was 146 mg/dL, and HbA1c was 6.5%, leading to a diagnosis of type 2 diabetes. At diagnosis, his height was 159.6 cm, his weight was 111 kg, and his BMI was 123%. Family history included a paternal grandfather with type 2 diabetes. Learning difficulties were suspected based on the medical history; therefore, a questionnaire was administered, which revealed high scores on the Parent-Interview ASD Rating Scale-Text Revision (PARS-TR) and ADHD rating scale (ADHD-RS) inattention domains, and difficulties with writing were acknowledged. On the Wechsler Intelligence Scale for Children, Fifth Edition (WISC-V), a significant discrepancy was noted between the Verbal Comprehension Index (VCI) and the Visual Spatial Index (VSI), with the VSI being markedly low. In the Kaufman Assessment Battery for Children, Second Edition (K-ABC2) achievement test, writing and arithmetic scales were markedly low. Based on these findings, the patient was diagnosed as having dysgraphia with visuospatial cognitive impairment, ASD, and inattentive-type ADHD.

Case 2

The patient was a 13-year-old male who had been overweight since the age of three years. After starting school, he became aware of learning difficulties and clumsiness in his hands. During a school health checkup, the patient tested positive for glucose in his urine, and blood tests revealed a random blood glucose level of 283 mg/dL and an HbA1c of 11.4%, leading to a diagnosis of type 2 diabetes. At diagnosis, his height was 162.3 cm, his weight was 105.6 kg, and his obesity level was 99.7%. Family history included type 2 diabetes and learning difficulties in his father. The questionnaire revealed inattention and difficulty in writing. On the WISC-V, his Full-Scale IQ (FSIQ) was at the lower limit of the average range, and his working memory index (WMI) was high, but his processing speed index (PSI) was significantly low. On the K-ABC2 achievement test, the writing scale score was significantly low. Based on these findings, the patient was diagnosed with dysgraphia, inattentive-type ADHD, and developmental coordination disorder (DCD).

Case 3

A 14-year-old male had experienced learning difficulties since the start of elementary school. At the same time, he developed a tendency toward obesity. Upon entering middle school, a urine test revealed positive glucose levels. Blood tests confirmed HbA1c of 7.7% and fasting blood glucose of 142 mg/dL, leading to a diagnosis of type 2 diabetes. At diagnosis, his height was 157.5 cm, his weight was 72.5 kg, and his BMI was 49.7%. The questionnaire revealed inattention and difficulty with writing. In the WISC-V, the FSIQ was at the lower limit of the average range, but there were deficits in the WMI and PSI. In the K-ABC2 achievement test, deficits were observed in the writing and mathematics scales. Based on these findings, the patient was diagnosed with dysgraphia, working memory impairment, inattentive-type ADHD, and DCD.

The findings of the questionnaire, WISC-V, and the K-ABC2 achievement scale for all cases are presented in Table 1. 

**Table 1:** Questionnaire, WISC-V, and K-ABC2 achivement scale findings PARS-TR: Parent-Interview ASD Rating Scale - Text Revision; ADHD-RS: Attention-Deficit/Hyperactivity Disorder-Rating Scale; WISC-V: Wechsler Intelligence Scale for Children - Fifth Edition; FSIQ: Full Scale Intelligence Quotient; VCI: Verbal Comprehension Index; VSI: Visual Spatial Index; FRI: Fluid Reasoning Index; WMI: Working Memory Index; PSI: Processing Speed Index; K-ABC2: Kaufman Assessment Battery for Children, Second Edition

		Case 1	Case 2	Case３	Cut-off value
PARS-TR (simplified version)	Preschool	9	0	3	7
	Present	9	0	5	9
ADHD-RS	Attention Deficit	19	14	17	
	Hyperactivity	6	1	0	
Reading and writing checklist	Reading	6	0	1	7
	Writing	11	8	9	7
WISC-Ⅴ	FSIQ	86	81	83	
	VCI	108	94	111	
	VSI	68	83	100	
	FRI	91	94	80	
	WMI	85	103	79	
	PSI	100	64	54	
K-ABC2 achievement scale	Comprehensive scale	92	90	79	
	Vocabulary scale	115	100	94	
	Reading scale	105	101	93	
	Writing scale	70	79	74	
	Arithmetic scale	77	84	71	

Executive function task

To evaluate executive functions, we administered Continuous Performance Tasks (CPT: Mogura-zu R) to measure inhibitory control and the Wisconsin Card Sorting Test (WCST, Keio version) to measure cognitive shifting (Table 2). In the CPT, Case 2 showed variability in reaction times and errors, and Case 3 also exhibited frequent omissions and emission errors. In the WCST, all patients demonstrated low achievement and high perseveration scores, indicating executive function impairment.

**Table 2:** Executive function test SD: standard deviation; WCST: Wisconsin Card Sorting Test

		Case 1	Case 2	Case 3
Continuous performance tasks (SD)	Correct answer rate	1.23	-6.41	-2.29
	Omission error	-0.5	8.75	1.25
	Commission error	-1.14	5.07	2.27
	Mean response time	-0.13	2.36	1.78
	Response time variability	-0.92	2.7	0.03
WCST (Keio.ver)	Achievement scores (SD)	1 (-0.50)	1 (-1.02)	2 (-0.51)
	Perseveration scores (SD)	17 (+1.25)	12 (+1.39)	6 (＋0.16)

## Discussion

The three children diagnosed with type 2 diabetes exhibited inattentive-type ADHD, dysgraphia, and impaired executive function. As mentioned earlier, 90% of children with type 2 diabetes are obese [1,2], and obesity is a major risk factor for type 2 diabetes in children. Furthermore, children with neurodevelopmental disorders have a high incidence of obesity, and it has been reported that approximately 40% of children with ADHD have a high obesity rate [4]. The suggested causes include poor dietary habits due to impulsivity or inattention, reduced physical activity (increased screen time), and genetic factors; however, the specific genes or mechanisms remain unclear [5]. Reduced activity in the prefrontal cortex and striatum, which are involved in executive function and motivation, has been reported in children with ADHD [6], and it is validated that attention, impulse control, and inhibitory control are impaired, often accompanied by executive dysfunction [7]. Therefore, in these three cases, the presence of executive function disorders in inattentive-type ADHD suggests a potential progression to obesity. Additionally, Cases 1 and 2 had family histories of diabetes, suggesting that genetic factors may have contributed to the progression of type 2 diabetes.

SLD is defined as a condition in which, despite average or above-average intellectual ability and no issues with the learning environment, an individual exhibits difficulties in acquiring specific skills, such as reading, writing, calculation, and numerical concepts. These are classified as reading, writing, and mathematics disorders [8]. In particular, writing kanji requires various elements such as visual perception, memory, visual representation, spatial construction ability, temporal processing, the act of writing, and concentration. A deficiency in any of these elements may lead to dysgraphia [9]. In ADHD, impaired concentration may contribute to dysgraphia, while in DCD, difficulties in writing may be a contributing factor [10,11]. In the present study, impaired visual perception in Case 1, coexisting DCD in Case 2, and impaired working memory combined with DCD in Case 3 were considered potential causes of dysgraphia.

Reports suggest a genetic link between type 2 diabetes and dyslexia. Genes associated with neuronal migration and dyslexia, such as ROBO1, DCDC2, DYX1C1, and KIA0319, KIA0319, which are linked to neural cell migration and dyslexia, and genes such as CTNNB1, TCF7L2, and KIF3A, which are associated with insulin secretion regulation and glucose transport and type 2 diabetes, are all involved in the WNT signaling pathway. This pathway regulates cell migration, cell fate determination, cell polarity, neural patterning, and organogenesis during embryonic development [12]. Furthermore, one study [13] indicates that 28% of children with type 2 diabetes also exhibit learning difficulties, suggesting a possible genetic relationship or association between SLD and type 2 diabetes. However, various factors contribute to dysgraphia, and its genetic basis remains unclear [14]. In Case 2, both type 2 diabetes and learning difficulties were observed in the father, but the genetic relationship is not clear, necessitating further research.

In these three cases, neurodevelopmental disorders were present before the onset of type 2 diabetes, presenting with executive function impairments. Obesity progression resulting from disrupted eating habits and lifestyle associated with these impairments is considered a major contributing factor to the development of type 2 diabetes. Furthermore, given the family history of type 2 diabetes in Cases 1 and 2, it is inferred that genetic factors, not just obesity, were involved in the onset of type 2 diabetes. However, the relationship between these factors and genetic factors remains unclear, and further research is necessary.

Additionally, pediatric patients with type 1 diabetes who also have neurodevelopmental disorders have been reported to have poorer glycemic control than those without such disorders [15]. Therefore, management by a diabetes specialist team is necessary. In type 2 diabetes, which is often accompanied by obesity, self-management of lifestyle habits is crucial in addition to diabetes treatment. However, patients with comorbid neurodevelopmental disorders may face similar difficulties in achieving glycemic control and maintaining appropriate treatment due to impaired executive function and insufficient understanding of the disease. In Japan, urine glucose testing during school urine screenings serves as a screening test for pediatric diabetes. If urine glucose is positive, further investigations, such as blood tests, are conducted to assess for diabetes. However, psychological testing, interviews regarding neurodevelopmental disorders or learning difficulties, and thorough evaluations are rarely performed. Therefore, when diagnosing and treating pediatric type 2 diabetes, the possibility of coexisting neurodevelopmental disorders must be considered. Based on the diagnosis, a tailored treatment plan should be developed. This approach can help improve long-term prognosis.

## Conclusions

We discussed three cases of type 2 diabetes mellitus with dysgraphia, inattentive-type ADHD, and executive dysfunction. The onset of type 2 diabetes mellitus in childhood may be associated with neurodevelopmental disorders and executive dysfunction. In addition, while it is well known that SLD often co-occurs with neurodevelopmental disorders such as ADHD and ASD, it has also become clear that SLD co-occurs with type 2 diabetes in children. Therefore, when diagnosing and supporting pediatric patients with type 2 diabetes, it is important to consider the possibility of comorbid SLD, in addition to ADHD and ASD.
